# Statement of Retraction: PSB0788 ameliorates maternal inflammation‐induced periventricular leukomalacia-like injury

**DOI:** 10.1080/21655979.2022.2163545

**Published:** 2023-01-03

We, the Publisher of the journal *Bioengineered*, have retracted the following article:

Yilu Li, Dan Wang, Zhuoyang Li & Zhi Ouyang . PSB0788 ameliorates maternal inflammation‐induced periventricular leukomalacia-like injury. Bioengineered. 2022; 13(4): 10224-10234. DOI: 10.1080/21655979.2022.2061296

Since publication, the authors have raised significant concerns about the reliability of the data presented in the article. As the Editor and Publisher also have concerns about the integrity of the reported results, all parties have agreed to retract the article to ensure correction of the scholarly record.

We have been informed in our decision-making by our policy on publishing ethics and integrity and the COPE guidelines on retractions.

The retracted article will remain online to maintain the scholarly record, but it will be digitally watermarked on each page as ‘Retracted’.

